# Editorial: Hip Arthroscopy

**DOI:** 10.3389/fsurg.2017.00029

**Published:** 2017-06-01

**Authors:** Shane J. Nho, Gift Ukwuani, Joshua D. Harris

**Affiliations:** ^1^Orthopedic Surgery, Rush University Medical Center, Chicago, IL, USA; ^2^Orthopedic Surgery, Houston Methodist Hospital, Houston, TX, USA

**Keywords:** hip arthroscopy, capsular management, femoroacetabular impingement, hip preservation surgery, cam deformity, pincer deformity, labral repair and reconstruction

**The Editorial on the Research Topic**

**Hip Arthroscopy**

Hip arthroscopy is a minimally invasive surgical procedure employed in the management of several hip pathologies. It is relatively novel procedure that has gained acceptance for the diagnosis and treatment of hip pathologies (Lee et al.; Kuhns et al.). Due to less morbidity, relatively shorter recovery time, and excellent outcomes, hip arthroscopy has become one of the fastest growing procedures performed in the field of orthopedic surgery (Lee et al.; Kuhns et al.).

The focus of this research topic is on the management of hip and pelvis pathologies. Femoroacetabular impingement (FAI) has only recently been described by Kuhns and colleagues as a clinical syndrome that results from abnormal osseous morphology of the proximal femur and acetabulum (Kuhns et al.). Numerous studies have cited that hip arthroscopy for the treatment of FAI may result in pain relief, improvement of activities of daily living, and return to sporting activities (Kuhns et al.).

The natural history of FAI is not well understood, but there are some patients that are at-risk of significant chondrolabral injury that may lead to early onset hip joint degeneration. Identification and treatment of hips at-risk may alter the natural progression of disease, but there is insufficient evidence currently in literature to suggest that surgical intervention in symptomatic or asymptomatic patients will prevent subsequent development of osteoarthritis.

Early diagnosis of FAI is dependent on an improved ability to identify abnormal morphology. Plain radiographs are the initial screening tests for FAI, but there are some patients with subtle pathomorphology. Levy and colleagues affirmed that CAM deformities are more common in males while pincer deformities are predominant in females (Levy et al.). They also reported that the CAM deformities in females may be more subtle than their male counterparts. Kuhn and others described the role of the capsule in hip pathology, but we still do not fully understand the differences in biologic and biomechanical profiles but likely contribute to altered kinematics of the hip (Strosberg et al.). Clinical and basic science studies have demonstrated that the capsule should be either repaired or plicated to prevent iatrogenic instability (Kuhns et al.).

Bittersohl and others described the use of magnetic resonance imaging as the most precise imaging modality in characterizing the extent of chondrolabral damage. The authors asserted that the integrity of the articular cartilage significantly affects the surgical decision to perform hip arthroscopy as well as the outcome of the procedure (Bittersohl et al.).

White and investigators described the labrum as a crucial contributor to hip joint stability by maintaining joint fluid pressurization and providing seal (White and Herzog). While the decision to perform labral debridement, repair, or reconstruction may depend on surgeon preference, the authors advocated that the indications for labral reconstruction include insufficient labral tissue or an irreparable labral tear (White and Herzog).

The relationship between FAI and surrounding neuromuscular dysfunction of the hip and pelvis has not been well defined and often exist together. Strosberg and colleagues explained that alterations in hip biomechanics in the setting of FAI lead to excessive strain on core musculature culminating in symptomatic core muscle injury (also known as athletic pubalgia or sports hernia) (Strosberg et al.).

Rehabilitation is a vital component of a successful outcome after hip arthroscopy. Grzybowski and others reported heterogeneity in rehabilitation after hip arthroscopy (Grzybowski et al.). Consequently, the authors believe that the development of evidence based protocols may provide consistency in the rehabilitation after hip arthroscopy.

In some cases, hip arthroscopy may not be able to address the deformity comprehensively, and open hip preservation techniques need to be utilized. Kuhns and colleagues reviewed the open techniques for the treatment of FAI in addition to developmental or acquired deformities of the acetabulum or femur (i.e., varus/valgus, torsion, or version), Legg–Calve–Perthes disease, and chronic slipped capital femoral epiphysis that may be more effectively managed by open procedures (Levy et al.).

The editors of the Hip Arthroscopy research topic believe that the content provides the most up to date information in a field that is rapidly evolving. We hope that you enjoy these articles and may stimulate further discussion and understanding of non-arthritic hip pathology.

## Author Contributions

SN, GU, and JH: research topic editor.

## Conflict of Interest Statement

The authors declare that the research was conducted in the absence of any commercial or financial relationships that could be construed as a potential conflict of interest.

